# Whole-genome sequencing for neonatal intensive care unit outbreak investigations: Insights and lessons learned

**DOI:** 10.1017/ash.2021.161

**Published:** 2021-06-24

**Authors:** Sarah E. Sansom, Latania K. Logan, Stefan J. Green, Nicholas M. Moore, Mary K. Hayden

**Affiliations:** 1 Division of Infectious Diseases, Department of Internal Medicine, Rush University Medical Center, Chicago, Illinois; 2 Division of Infectious Diseases, Department of Pediatrics, Rush University Medical Center, Chicago, Illinois

## Abstract

Infectious diseases outbreaks are a cause of significant morbidity and mortality among hospitalized patients. Infants admitted to the neonatal intensive care unit (NICU) are particularly vulnerable to infectious complications during hospitalization. Thus, rapid recognition of and response to outbreaks in the NICU is essential. At Rush University Medical Center, whole-genome sequencing (WGS) has been utilized since early 2016 as an adjunctive method for outbreak investigations. The use of WGS and potential lessons learned are illustrated for 3 different NICU outbreak investigations involving methicillin-resistant *Staphylococcus aureus* (MRSA), group B *Streptococcus* (GBS), and *Serratia marcescens*. WGS has contributed to the understanding of the epidemiology of outbreaks in our NICU, and it has also provided further insight in settings of unusual diseases or when lower-resolution typing methods have been inadequate. WGS has emerged as the new gold standard for evaluating strain relatedness. As barriers to implementation are overcome, WGS has the potential to transform outbreak investigation in healthcare settings.

The highest burden of hospital-acquired infections (HAIs) is observed in intensive care units (ICUs).^1^ Infants admitted to neonatal ICUs (NICUs) are particularly vulnerable to infectious complications. Preterm infants often have impaired immune function and skin integrity, extensive exposure to antibiotics, undergo invasive or surgical procedures, have prolonged indwelling devices, and have multiple contacts with healthcare providers.^2^ Infection outbreaks can be devastating in this population, and they are associated with high morbidity, cost, and mortality.^3^ Rapid recognition and response to outbreaks is essential to protecting this vulnerable population.

Whole-genome sequencing (WGS) has emerged as a powerful tool in epidemiologic and outbreak investigation. WGS provides superior resolution to discriminate between genetically similar organisms, even among highly related lineages,^4^ and it has been used to investigate and terminate many outbreaks.^5^ WGS provides accurate identification of the pathogen and identifies sequence differences to the level of single-nucleotide variants (SNVs). Additionally, WGS uniquely allows for longitudinal analysis of samples due to the expected accumulation of genetic mutations over time, allowing identification of chains of transmission in addition to clusters.^5^

In our institution, WGS has been used since early 2016 as an adjunctive method to pulsed-field gel electrophoresis (PFGE) for outbreak investigations. However, as the following cases illustrate, PFGE may lack adequate discriminatory power in certain situations. WGS has provided real-time, critical information for a deeper understanding of outbreak epidemiology in the NICU. Here, we describe selected cases and insights from our single-center experience with WGS as a tool for NICU outbreak investigation.

## Materials and methods

Rush University Medical Center (Rush) is a 650-bed tertiary-care, academic institution in Chicago, Illinois. The Renée Schine Crown NICU at Rush is a 40,000-square-foot, level 3, labor-and-delivery NICU with 3 pods and 60 single beds. During the study period, clinical and surveillance cultures from infants admitted to the NICU were monitored by infection control personnel. Samples were collected and cultured according to routine clinical microbiologic practices and standards^6^ at Rush Medical Laboratories, which is certified by Clinical Laboratory Improvement Amendments (CLIA) and accredited by The College of American Pathologists (CAP). Bacterial isolates from cultures were submitted to the Genome Research Core (GRC) and Research Informatics Core (RIC) at the University of Illinois—Chicago for nucleic acid extraction, library preparation, WGS, and genomic analysis, as described previously.^7^ Informed consent was not required because clinical samples were collected during routine clinical care and investigations were performed as part of infection control management. The project was waived from review by the institutional review board.

Environmental samples were collected by personnel from Hygieneering (Willowbrook, IL) and were submitted to EM Lab P&K (Marlton, NJ) for semiquantitative bacterial culture. Surface samples were collected with moistened sterile swabs, and water samples were collected in sterile glass or plastic bottles. Samples were inoculated on tryptic soy agar (TSA) plates. Bacterial growth was identified according to routine microbiologic procedures.^8^

### *Clinical vignette 1: Methicillin-Resistant* Staphylococcus aureus *(MRSA)*

**Fig. 1.**
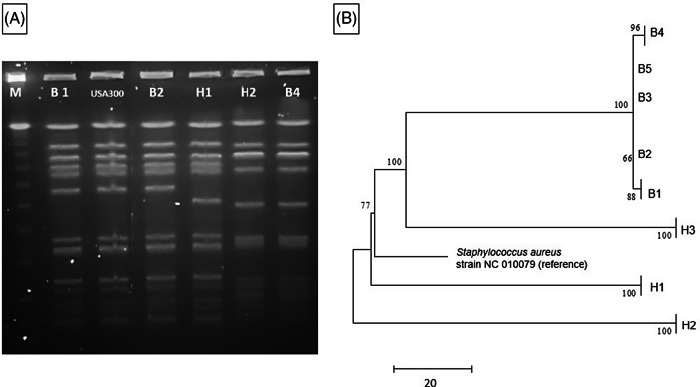
MRSA outbreak investigation. (A) PFGE of MRSA isolates from healthcare workers and infants. Total genomic DNA of MRSA isolates was digested using *SmaI* restriction enzyme and resolved in 1% agarose in 0.5× TBE buffer (pulse time, 1–50 seconds; run time, 21 hours). Selected infant isolates (B1, B2, and B4) showed similar banding patterns to HCP isolates (H1 and H2) (all USA 300). Molecular weight marker (M) is shown in the first column. (B) Neighbor-joining phylogenetic tree based on whole-genome sequencing analysis of MRSA isolates from the NICU outbreak. Genomic sequence data were processed using the software package SPANDx^37^ to generate a variant matrix. This matrix was used to create a bootstrapped phylogenetic tree within the software package MEGA.^38^ The scale bar represents genetic distances based on number of nucleotide differences, and the numbers at nodes represent bootstrap support (1,000 iterations; only nodes with >70% support are indicated). Isolates were compared to a reference MRSA USA 300 genome (GenBank accession no. NC 010079). Isolates collected from the infants (“B” isolates) were highly similar; at most, 5 SNVs were detected between infant isolates. HCP isolates (H1 and H2) were highly divergent from the infant isolates, and unrelated to each other. One HCP isolate (H3) shared a common ancestor more recently with the infant isolates than the other HCP isolates but differed from the infant isolates by >100 nucleotides, indicating that H3 was not the source of the outbreak. Note. HCP, healthcare personnel; MRSA, methicillin-resistant *Staphylococcus aureus*; SNV, single nucleotide variant.

A clinical endotracheal aspirate sample from a 6-month-old infant (B1) in the NICU grew MRSA during an evaluation for lower respiratory tract infection (index case). Subsequently, all patients in the same NICU pod underwent MRSA surveillance by anterior nares swab culture. Four infants (B2, B3, B4, and B5) in nonadjacent rooms had asymptomatic colonization with similar USA 300 strain types upon PFGE analysis (Table 1).^9^ Total unit surveillance was done next, which identified 1 additional infant (B6) in a different pod who had MRSA colonization. Notably, this infant was previously known to carry a different strain of MRSA as determined by earlier PFGE testing. Next, screening of all 291 NICU healthcare personnel (HCP) by anterior nares swab identified 5 HCP (1.7%) as MRSA carriers; all 3 of these isolates (H1, H2, and H3) were similar by PFGE to the MRSA isolates in the infant cluster (ie, they matched a USA 300 pattern). All MRSA-colonized staff were referred for decolonization. Figure 1A shows a PFGE analysis of representative MRSA isolates and demonstrates similar banding patterns between select infant and HCP isolates,^9^ suggesting a common source or cross transmission of the outbreak strain.

**Table 1.** Characteristics of Infants with MRSA Isolates

Clinical Data	Infant Information				
	B1	B2	B3	B4	B5
GA at birth, weeks	35	31	34	28	24
Birth weight	LBW	LBW	NBW	VLBW	LBW
GA at detection, weeks	64	33	59	34	34
Specimen type	Endotracheal aspirate	Surveillance	Surveillance	Surveillance	Surveillance
Surgical procedures	Yes	None	Yes	None	None
Ventilator	Yes	No	Yes	No	No
Invasive devices	CVC, G-tube	None	J-tube	None	None

Note. GA, gestational age; LBW, low birth weight; VLBW, very low birth weight; NBW, normal birth weight; CVC, central venous catheter; G-tube, gastrostomy tube; J-tube, jejunostomy tube.

The 5 infant isolates and 3 HCP MRSA isolates with similar PFGE patterns were submitted for WGS, with a turnaround time of 4.5 days for extraction, library preparation, sequencing, data processing, and phylogenetic analysis (Fig. 1B). The infant isolates were highly similar, with 5 nucleotide differences at most. However, 2 HCP isolates (H1 and H2) were highly divergent from the infant isolates and were not closely related to each other. Isolate H3 shared a more recent common ancestor with the infant isolates than did the other HCP isolates, but this isolate had >100 nucleotide differences, indicating that H3 was also unlikely to be the source of the infant outbreak. Previous MRSA outbreak investigations have proposed SNV cutoffs (range, ≤22 to <60 SNVs) as indicative of isolate genomic relatedness.^10^

The WGS results suggested that the cluster of MRSA colonization and infection was likely due to cross transmission within the NICU via contaminated HCP hands and was not due to a point-source outbreak related to a colonized HCP. However, a point source outbreak related to an undetected healthcare worker or environmental reservoir could not be ruled out. The rapid turnaround time of WGS allowed rapid implementation of measures to reduce cross transmission. The outbreak resolved with staff education and reinforcement of routine infection control practices including patient cohorting and enhanced hand hygiene compliance.

MRSA can cause a wide variety of infectious complications in neonates; the most common is late-onset sepsis. MRSA infection is associated with increased morbidity and mortality in premature and low-birth-weight neonates.^12,13^ The most common site of colonization is the anterior nares, but MRSA can also be detected in the umbilicus, axilla, groin and perineum.^12^ Colonization is the most important step preceding development of MRSA infection; ∼1 in 5 neonates colonized with MRSA may develop an infection, with rates even higher in premature infants.^12^ Despite aggressive infection control measures, MRSA remains endemic in some NICUs.
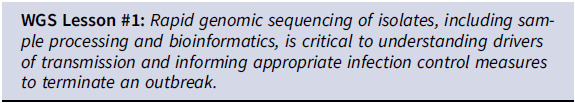



### *Clinical vignette 2: Group B* Streptococcus

**Fig. 2.**
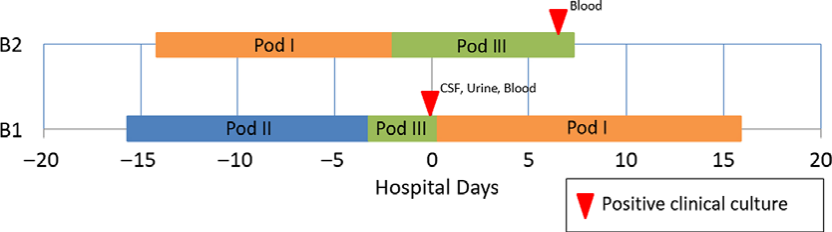
Epidemiologic map of 2 patients with late-onset disease group B *Streptococcus* (GBS). The infants were located in separate NICU pods at the time of positive clinical cultures, but previously shared a 2-day overlap period in the same NICU pod. WGS showed that the isolates were highly similar. In total, results of the outbreak investigation suggested horizontal transmission.

A 15-day-old premature infant in the NICU (B1) developed group B streptococcal (GBS) sepsis with meningitis. GBS carriage in the mother was known from rectovaginal culture at delivery. Seven days later, a second premature infant (B2), whose mother did not have any history of GBS colonization, also developed sepsis with GBS. (Table 2) The infants were located in separate NICU pods at the time of positive clinical cultures. However, a review of room history revealed that there was a 2-day period before the first positive GBS clinical cultures during which the infants were located 5 rooms apart in the same NICU pod (Fig. 2). No other clinical cases of GBS were identified, and no surveillance of babies for GBS colonization was performed. No additional clinical cases of GBS were identified in the following 3 months.

**Table 2.** Characteristics of Infants with GBS Isolates

Infant Identification	Weeks GA	BW	Time From Birth to GBS	Mother GBS Status	CS	Ventilator	Clinical Syndrome	Clinical Specimens Positive
B1	32	LBW	15 days	+	No	No	Sepsis, bacteriuria, meningitis	Urine, blood, CSF
B2	32	LBW	20 days	−	Yes	No	Sepsis	Blood

Note. GA, gestational age; BW, birth weight; LBW, low birth weight; GBS, group-B *Streptococcus*; CS, cesarean section; NICU, neonatal intensive care unit.

Due to the unusual nature of concurrent patients with late-onset GBS disease, both isolates were submitted for WGS, as described above. Genomes were highly similar, with only 3 nucleotide differences. This result was consistent with a limited cluster of GBS transmission in the NICU with spontaneous resolution of the outbreak. GBS disease is largely clonal,^14^ and outbreaks may be frequently missed due to prolonged intervals between cases within clusters.^15^ A population-based evaluation of 754 unique invasive GBS isolates from the United Kingdom and Ireland suggested the use of ≤5 SNV differences to define invasive GBS clusters.^15^ Identification of a possible nosocomial link in this outbreak may provide key insights for future research efforts.

Invasive GBS infection is an important cause of neonatal sepsis; bacteremia and meningitis are the most common presentations in late-onset disease. Early-onset disease occurs at 0–6 days of life, while late-onset disease occurs between 7 and 89 days. Early-onset disease results from vertical transmission during the intrapartum period. However, the epidemiology of late-onset disease remains incompletely understood,^16^ with high morbidity and mortality in preterm infants.^17,18^ Although early-onset disease has been decreasing with implementation of intrapartum antibiotic prophylaxis, the incidence of late-onset disease has remained stable over the past decade.^17^

Few modern outbreaks of late-onset GBS disease that used genomic methods for investigation are available in the literature. Juaneikaite et al^19^ described genomic analysis of 12 late-onset invasive GBS cases that revealed 4 clusters over 2 years. A common source was supported by the low number of observed SNVs (0–5 differences) between isolates. Although a clear source was not identified, a potential breakdown of multiple hygiene practices (eg, breast-pump hygiene) was suspected as the cause. Several additional clusters identified by Collin et al^15^ through population-based genomic epidemiology further supports the possibility of horizontal transmission. The authors hypothesized that clusters of infection could be driven by environmental contamination or by transmission via intermediary staff or equipment. In the current investigation, WGS results provided support for the hypothesis that late-onset GBS disease may be the result of nosocomial transmission.
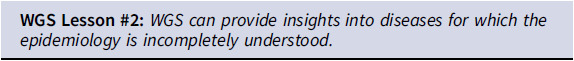



### *Clinical vignette #3:* Serratia marcescens

A preterm infant in the NICU (B1) grew *Serratia marcescens* from an eye-discharge culture. Seven days later, 2 additional infants (B2 and B3) grew *S. marcescens* from respiratory specimens. On day 8, a fourth infant (B4) grew *S. marcescens* from an endotracheal aspirate. On day 10, perirectal screening of NICU patients identified 3 more asymptomatically colonized infants (B5, B6, and B7) (Table 3). Most infants were located within close spatial proximity in the NICU but had not been housed in the same rooms. Four infants (B1, B5, B6, and B7) were located in adjacent rooms in 1 pod. Three infants (B2, B3, and B4) were located in adjacent rooms in a separate pod. HCP screening was not performed.

**Table 3.** Characteristics of Infants With *Serratia marcescens* Isolates

Clinical Data	Infant Information						
	B1	B2	B3	B4	B5	B6	B7
Weeks GA at birth	26	27	25	27	26	29	29
Birth Weight	VLBW	VLBW	VLBW	VLBW	VLBW	LBW	VLBW
Weeks GA at detection	32	35	29	31	37	33	36
Specimen	Wound/ eye	Tracheal aspirate	Sputum	Tracheal aspirate	Perirectal screen	Perirectal screen	Perirectal screen
Day of outbreak identified	1	7	7	8	10	10	10
Ventilator	No	Yes	Yes	Yes	No	No	No

Note. GA, gestational age; VLBW, very low birth weight; LBW, low birth weight.

**Fig. 3.**
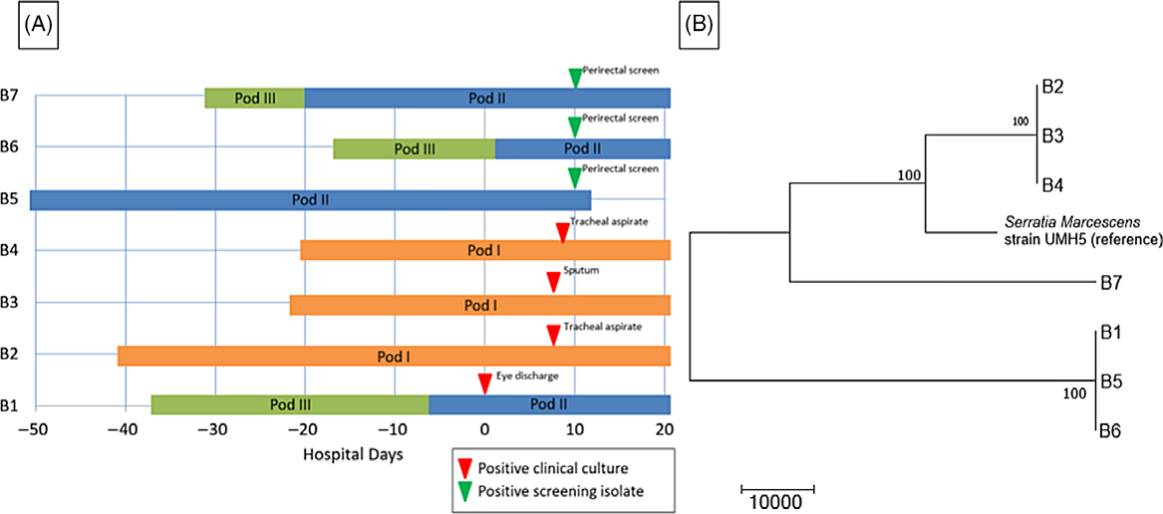
*Serratia marcescens* outbreak investigation. (A) Epidemiologic map of 7 patients with *S. marcescens*. Clinical and screening isolates are shown. Most of the patients were housed within close spatial proximity to each other in the NICU. Infants B1, B5, B6, and B7 were located in adjacent rooms in pod II. Infants B2, B3, and B4 were located in adjacent rooms in pod I. (B) Neighbor-joining phylogenetic tree based on whole-genome sequencing analysis of *Serratia marcescens* isolates from the NICU outbreak. The phylogenetic tree was created as described previously. The scale bar represents genetic distances based on number of nucleotide differences, and the numbers at nodes represent bootstrap support (1,000 iterations; only nodes with >70% support are indicated). Isolates were compared to a reference *S. marcescens* genome (GenBank accession no. CP018917). Two separate clusters of 3 organisms each were identified, with the seventh organism not closely related to either group (B7). The genomes of the organisms within each cluster were identical or nearly identical. The genomes of isolates of cluster 1 (comprised of isolates B2, B3, and B4) were identical without detectable nucleotide differences. The genomes of isolates of cluster 2 (comprised of B1, B5, and B6) had at most 1 nucleotide mismatch between isolates. The clusters were highly distinct from one another, with tens of thousands of nucleotide differences.

The WGS revealed 2 separate clusters of highly related organisms (Fig. 3). The genomes of isolates of the first cluster (which included isolates B2, B3 and B4) were identical without detectable nucleotide differences. The genomes of isolates of the second cluster (including B1, B5, and B6) had zero or 1 nucleotide difference between isolates. The clusters were distinct from one another, with tens of thousands of nucleotide differences. The clusters were spatially related: infants (B1, B5, and B6) in one pod had the same strain and infants (B2, B3, and B4) in a second pod share a different strain. Infant B7 in the second pod was colonized with a separate unrelated strain of *S. marcescens*. In the literature, proposed SNV cutoff thresholds for relatedness of isolates for *S. marcescens* are limited. For example, 25 SNVs was the proposed cutoff in several publications, but smaller SNV cutoffs have been proposed in other outbreak reports.^20,21^

The NICU underwent deep cleaning on day 7 of the outbreak. Black-light inspections of high-touch surfaces were conducted to monitor adequacy of cleaning. Extensive environmental sampling was performed on day 10 of the outbreak including public and staff restrooms (eg, shower head, water from faucet, hand sink, and hand dryer), patient room (eg, hand sink, sink handles, nasal cannula, IV pump, bed humidifier and reservoir, bedside cabinet, bed fan and underside, bed heating element, mattress cover, bassinet, and curtain), hallway, and common areas (eg, scrub sink, alcohol pump, soap dispenser, and water fountain), respiratory therapy areas (eg, procedure hand sink, clean room door handle, clean room hand sink, and procedure-room dirty sink), and other high-touch items (eg, keyboards, handheld portable phones, interior/exterior door handles, bar code scanner, and lotion bottle). Although several gram-negative bacteria were isolated, *S. marcescens* was not detected in any of the 46 environmental samples that were collected.

Routine infection control measures were reinforced, including proper glove use and requiring “bare below the elbows” by HCP. Routine black-light inspections of high-touch surfaces continued. New sink drain covers were installed (Biodome, Boehringer Laboratories, Pheonixville PA), and a new quaternary disinfectant sink-flushing protocol was implemented (Virex II 256, Diversey, Fort Mill, SC). Examination of clinical equipment identified several ventilators with components that had missed preventive maintenance; any outstanding maintenance was promptly performed; and revised preventive maintenance schedules were implemented. Additionally, the cleansing procedure was revised, and 5 Giraffe beds were removed. No subsequent cases of *S. marcescens* were detected.

*Serratia marcescens* is the third most commonly identified microbial etiology of NICU outbreaks in published literature.^3^ It can cause a wide variety of clinical syndromes, including bacteremia, meningitis, pneumonia, conjunctivitis, and urinary tract infections.^22^ *S. marcescens* is pervasive in the hospital environment and can also cause early neonatal gastrointestinal colonization.^23^ A number of outbreak sources have been described, including medical equipment, food, baby bottles, intravenous or liquid medications, and the environment (eg, sink traps, liquid soap, respiratory oscillator).^24–27^ However, environmental sampling during outbreak investigation is rarely revealing,^1,28^ and the source of *S. marcescens* NICU outbreaks is not identified in 60% of published reports.^24^ Additionally, it is common to identify >1 strain of *S. marcescens* during a single or sequential outbreak.^23,29^

In this case, WGS was useful to identify 2 separate clusters of *S. marcescens* in the NICU. Utilizing traditional epidemiologic investigation methods, only a single cluster would have been suspected. Genomic analysis combined with close spatial proximity suggested transmission from 2 separate environmental sources and by HCP hand transmission between infants in close spatial proximity.

## Discussion

WGS has emerged as the gold standard for evaluation of strain relatedness during bacterial outbreak investigations. WGS can identify transmission events that are missed by less discriminatory methods,^30^ and it can expand our ability to detect unsuspected nosocomial transmissions, as well as to identify chance clusters.^31,32^ WGS has the potential to provide pertinent clinical information regarding antimicrobial resistance and virulence genes that may not be detected by phenotypic or targeted molecular testing.^4^ The robust capacity and scale of WGS has been useful to document transmission both within and between hospitals, as well as on an intercontinental scale.^33^ WGS is replacing other molecular genomic methods as the primary molecular subtyping tool. For example, the Centers for Disease Control and Prevention (CDC) and public health laboratories have transitioned foodborne pathogen strain typing from PFGE to WGS with early evidence of improved precision.^34^ However, WGS alone should not replace traditional surveillance and epidemiologic investigation because outbreak detection is highly complex, requiring WGS interpretation and evaluation of potential points of transmission in a healthcare facility.

Peacock et al^31^ suggested a “sequence first” approach to outbreak investigation. This strategy may involve routine genomic surveillance to provide early warning of transmission events or outbreaks, thus allowing for earlier recognition and implementation of control measures. Prospective surveillance with WGS has been shown to be feasible and may result in cost savings.^10,32,35^

Although there have been significant advances in the accessibility of WGS in the past decade, several challenges remain in its implementation as a routine tool for outbreak investigation. The largest hurdles are turnaround time, accessibility, cost, and standardization. Methods for library preparation are becoming more rapid. Instruments with rapid (<1 day) sequencing capabilities are now available, and turnaround times from sample collection to analyzed results have decreased to as little as 2 days.^31,32,36^ The availability of benchtop instruments with short run times, such as the Oxford Nanopore MinION and the Illumina iSeq and MiniSeq sequencers, represent particular advances in accessibility of WGS within individual healthcare facilities.^31^ However, interpretation of data remains a roadblock in facilities without bioinformatics expertise, which will likely need to be overcome by availability of complete analysis software.^4,10^

In conclusion, bacterial outbreaks cause significant morbidity and mortality in susceptible neonates admitted to the NICU. Prompt identification of the outbreak source with implementation of infection control measures are critical to limit further transmission. Older molecular typing methods (ie, PFGE) may fail to adequately discriminate between isolates and may lead to erroneous inferences about strain relatedness. WGS has become the new gold standard for strain typing in outbreak investigation because it provides superior resolution to determine strain relatedness. We have described 3 outbreaks with different organisms (MRSA, GBS, and *Serratia marcescens*) in a single NICU in which WGS proved to be a useful tool. As barriers to its use decrease, WGS is revolutionizing outbreak investigation, and it is expected to become more accessible as cost and turnaround times decrease. Emerging applications of WGS include primary genomic surveillance, which could identify potential transmission earlier to reduce morbidity and mortality associated with outbreaks.
