# Whole-genome sequencing for neonatal intensive care unit outbreak investigations: Insights and lessons learned – ADDENDUM

**DOI:** 10.1017/ash.2021.182

**Published:** 2021-08-03

**Authors:** Sarah E. Sansom, Latania K. Logan, Stefan J. Green, Nicholas M. Moore, Mary K. Hayden

In the above article^1^, WGS Lesson #3 was originally omitted; it reads:

WGS Lesson #3: WGS provides high resolution for strain typing in outbreak investigations and can provide support for transmission clusters that would have otherwise been miscategorized
